# SNPs in *SNCA*, *MCCC1*, *DLG2*, *GBF1* and *MBNL2* are associated with Parkinson's disease in southern Chinese population

**DOI:** 10.1111/jcmm.15508

**Published:** 2020-07-11

**Authors:** Aonan Zhao, Yuanyuan Li, Mengyue Niu, Guanglu Li, Ningdi Luo, Liche Zhou, Wenyan Kang, Jun Liu

**Affiliations:** ^1^ Department of Neurology and Institute of Neurology Ruijin Hospital Affiliated with the Shanghai Jiaotong University School of Medicine Shanghai China; ^2^ Department of Neurology Ruijin Hospital North Affiliated to Shanghai Jiaotong University School of Medicine Shanghai China; ^3^ CAS Center for Excellence in Brain Science and Intelligence Technology Ruijin Hospital Affiliated to Shanghai Jiaotong University School of Medicine Shanghai China; ^4^ Department of Neurology RuiJin Hospital/Lu Wan Branch, School of Medicine, Shanghai Jiaotong University Shanghai China

**Keywords:** *DLG2*, *GBF1*, *MBNL2*, *MCCC1*, Parkinson's disease, *SNCA*

## Abstract

Numerous single nucleotide polymorphisms (SNPs), which have been identified as susceptibility factors for Parkinson's disease (PD) as per genome‐wide association studies, have not been fully characterized for PD patients in China. This study aimed to replicate the relationship between 12 novel SNPs of 12 genes and PD risk in southern Chinese population. Twelve SNPs of 12 genes were detected in 231 PD patients and 249 controls, using the SNaPshot technique. Meta‐analysis was used to assess heterogeneity of effect sizes between this study and published data. The impact of SNPs on gene expression was investigated by analysing the SNP‐gene association in the expression quantitative trait loci (eQTL) data sets. rs8180209 of *SNCA* (allele model: *P* = .047, OR = 0.77; additive model: *P* = .047, OR = 0.77), rs2270968 of *MCCC1* (dominant model: *P* = .024, OR = 1.52), rs7479949 of *DLG2* (recessive model; *P* = .019, OR = 1.52), rs10748818 of *GBF1* (additive model: *P* < .001, OR = 0.37), and rs4771268 of *MBNL2* (recessive model: *P* = .003, OR = 0.48) were replicated to be significantly associated with the increased risk of PD. Noteworthy, a meta‐analysis of previous studies suggested rs8180209, rs2270968, rs7479949 and rs4771268 were in line with those of our cohort. Our study replicated five novel functional SNPs in *SNCA*, *MCCC1*, *DLG2*, *GBF1* and *MBNL2* could be associated with increased risk of PD in southern Chinese population.

## 1 INTRODUCTION

Parkinson's disease (PD) is the second most common neurodegenerative disease, which is diagnosed based on the motor signs of bradykinesia, rigidity and tremor.^1^ Although the effective symptomatic therapies for both motor and nonmotor manifestations of PD exist, there are no preventive or neuroprotective treatments available, which mean that the progressive decline of PD is inevitable. The pathogenesis of PD has been linked to a loss of dopaminergic neurons in substantia nigra as well as pathologic α‐synuclein aggregation.^2^ However, the aetiology of sporadic PD is still unclear. Theories suggest that it might be caused by the confounding influence of genetic and environmental risk factors, such as toxins and pesticide exposure.^3^

An interesting study of the heritability of PD risk, involving over 500 families, revealed that in up to 60% of idiopathic PD patients the phenotype could be explained by genetic factors.^4^ The identification of patients with PD risk alleles may be helpful for early diagnosis, further paving the way for the personalized medicine.^5^ The first genome‐wide association study (GWAS) confirmed the causal genes *SNCA*, *PARK16*, *LRRK2* and *BST1* as risk genes for PD.^6^ Subsequent GWASs, in increasingly larger patient‐control cohorts, and meta‐analyses not only confirmed candidate gene‐based and former GWAS associations but also revealed additional risk genes like *RIT2*, *GCH1* and *STK39*.^7^, ^8^ Since many of the associated GWAS SNPs reside in noncoding regions and large numbers of individuals are required to be analysed, integrative analysis that combines both DNA sequencing and gene expression would accelerate the identification and functional characterization of the biological variants and PD‐related genes.^9^

Recently, the two GWAS results in PD identified several new PD risk loci.^10^, ^11^ However, because of differences in allele frequencies among different ethnicities, the analysis is not directly applicable to the Chinese population. As a result, the association of these novel candidate loci with PD in the southern Chinese population remained unclear. In this study, we aimed to explore the relationship between these newly characterized risk alleles and PD in southern Chinese population compared with previous GWAS studies and further discuss the potential effect of the susceptible loci on the respective gene expression by the expression quantitative trait loci (eQTL) analysis.

## 2 METHODS AND MATERIALS

### 2.1 Study population

A total of 231 PD patients were recruited from September 2017 to July 2019 from the outpatient clinic at the department of neurology, Ruijin Hospital affiliated to Shanghai Jiaotong University School of Medicine. PD was diagnosed by the movement disorder specialists using the MDS diagnostic criteria.^12^ A total of 249 healthy control (HC) subjects were recruited and examined by the movement disorder specialists to exclude any possibility of PD. Patients with the medical history of other neurodegenerative diseases and/or inflammatory‐, drug induced‐, vascular‐ or toxin‐induced parkinsonism were all excluded. Based on the diagnostic age, PD patients were divided into two groups: late‐onset PD (LOPD) group and an early‐onset PD (EOPD) group. All patients with the first diagnostic age of PD more than 45 years were placed in the LOPD group,^13^ and remaining were placed in the EOPD group. Mild PD was defined when Hoehn‐Yahr staging was below 2.5 after assessment.^14^ Patients with relatives who have PD (within the last three generations) were regarded as familial PD patients. This study was approved by the Ethics Committee of the Ruijin Hospital affiliated to the Shanghai Jiaotong University School of Medicine, and all participants provided written informed consent.

### 2.2 DNA extraction and genotype analysis

Two millilitre of venous blood sample was collected in EDTA anti‐coagulation tubes from PD patients and healthy controls. The phenol‐chloroform‐isopropyl alcohol method was used to extract genomic DNA. Polymer chain reaction and extension primers were designed using Primer5 software (version 5.0; PREMIER Biosoft International). The SNaPshot technique was used to genotype SNPs. The following SNPs were tested: rs8180209, rs2270968, rs7479949, rs10748818, rs61169879, rs9261484, rs4771268, rs11610045, rs2248244, rs12528068, rs2904880 and rs1450522. Details of primers are described in Table S1.

### 2.3 Expression quantitative trait loci analysis

The potential functional impact of validated SNPs on gene expression was evaluated by analysing gene‐SNP association in eQTL studies with two different databases: the Braineac eQTL data set and the GTEx (Genotype Tissue Expression Project) database. The Braineac eQTL data set, a public database developed by the UKBEC (the UK Brain Expression Consortium, UKBEC), integrates genotypes and gene expression data from 134 human brain samples of 10 brain regions,^15^ while the GTEx database integrates genotypes and gene expression data of various tissues from 544 donors with different pathological diseases.^16^

### 2.4 Construction of luciferase reporter gene vectors and dual‐luciferase reporter assays

The *GBF1* promoter plasmid containing the A or G allele at rs10748818 was amplified from the genomic DNA of HC, using primers containing BglII in the forward primer and HindIII in the reverse primer for cloning (forward: 5′‐GAAGATCTACTGCTCTAGTCCTGTGGGT‐3′ and reverse: 5′‐CCCAAGCTTCATTGCAACCCTGAGATACCCC‐3′). Jurkat cells (human T lymphocyte cells) and SH‐SY5Y (human neuroblastoma cells) were plated into 24‐well culture plates 24 hours prior to transfection and 490 ng polymorphism plasmid or pGL3‐basic empty plasmid (as a negative control) was transfected using Lipofectamine 3000 (Invitrogen), with 10 ng Renilla pRL‐TK plasmid (Promega) co‐transfected as a normalizing control. After 24 hours, cells were rinsed with PBS and harvested with Passive Lysis buffer (Promega). Transcriptional activity was determined using the Dual‐Luciferase Reporter Assay System (Promega) on a Synergy H4 Hybrid Microplate Reader (BioTek). For each plasmid construct, four independent transfection experiments were carried out and readings were taken in duplicate. The transcriptional activities were reported as relative luciferase activities, which was the ratio of firefly luciferase activities over renilla luciferase activities.

### 2.5 Statistical analyses

Data were analysed by the SPSS software version 25.0 (SPSS Inc). A *t* test was used to compare the differences in age between PD patients and controls. A chi‐square test was used to study the differences in the sex proportions, the discrepancy in allele and genotype frequencies and to test the Hardy‐Weinberg equilibrium (HWE). Logistic regression analysis was used to calculate the risk analysis of each SNP in dominant, recessive and additive models after adjusting for age and gender. The genetic power of each SNP was calculated using Power and Sample Size software (version 3.1.6).^17^ Multiple tests were performed using the Bonferroni correction method. Meta‐analysis was performed using Review Manager 5.2 for Windows. Linkage disequilibrium (LD) linkage analysis was performed on the platform of SHEsis.^18^ *Q*‐statistics and *I* ^2^ were used for assessing the heterogeneity. Statistical significance was taken as two‐sided *P* < .05.

## 3 RESULTS

### 3.1 Demographic and clinical characteristics of the study population

The demographic and clinical characteristics of 231 PD patients and 249 HC subjects are shown in Table 1. No significant difference could be observed in the age and the gender between the PD and HC groups. Of all patients with PD, 54.11% were male, 77.06% had mild PD, 8.66% had familial PD, and the average age of PD was 63.96 ± 8.46 years (Table 1).

**TABLE 1:** Demographic data of PD and controls

	PD patients (N = 231)	Controls (N = 249)	*P*‐value
Gender, female, N (%)	106 (45.89)	105 (42.17)	.525
Age, mean (SD), y	63.96 (8.46)	65.37 (9.09)	.086
Mild PD, N (%)^a^	178 (77.06)	/	/
Familial PD, N (%)	20 (8.66)	/	/
EOPD, N (%)	36 (15.59)	/	/
Gender, female, N (%)^b^	15 (41.67)	/	/
Mild PD, N (%)^b^	23 (63.89)	/	/
Familial PD, N (%)^b^	6 (16.67)	/	/
LOPD, N (%)	195 (84.42)	/	/
Gender, female, N (%)^c^	91 (46.67)	/	/
Mild PD, N (%)^c^	155 (79.49)	/	/
Familial PD, N (%)^c^	14 (7.19)	/	/

Abbreviations: EOPD, early‐onset Parkinson's disease; LOPD, late‐onset Parkinson's disease; PD, Parkinson's disease; SD, standard deviations.

^a^ Assessed by Hoehn‐Yahr Stage.

^b^ Referred to EOPD.

^c^ Referred to LOPD.

### 3.2 Analysis of genotypic and allele frequency in PD

For all the SNPs, genotype distributions were in the HWE (Table S2). The minor allele frequencies and genotype frequencies of all these SNPs are listed in Table S2, and the SNPs found to be significantly associated with PD are listed in Table 2. The following models were found to be associated with the PD, both allele and additive model of the rs8180209 of *SNCA* (allele model: *P* = .047, OR = 0.77; additive model: *P* = .047, OR = 0.77); the dominant model of the rs2270968 of *MCCC1*, with (*P* = .023, OR = 1.52) or without (*P* = .024, OR = 1.52) adjusting the age and gender; and the recessive model of the rs7479949 of *DLG2* (*P* = .019, OR = 0.26). After the Bonferroni correction, only two SNPs showed significant association with the increased risk of PD: rs10748818 of *GBF1* (additive model: *P* < .001, OR = 0.37) and rs4771268 of *MBNL2* (recessive model: *P* = .003, OR = 0.48). Furthermore, the rs4771268 of *MBNL2* also revealed significant association with PD under both allele and additive models (allele model: *P* = .011, OR = 0.72; additive model: *P* = .015, OR = 0.73). Additionally, the five SNPs were revalidated in another independent cohort with 191 PD patients and age‐ and gender‐matched 173 controls. The significance of the rs8180209 of *SNCA,* rs10748818 of *GBF1* and the rs4771268 of *MBNL2* persisted under allele models which supported our original results (Table S3).

**TABLE 2:** Association of SNPs of candidate genes and odds ratio to PD risk

Candidate gene	SNP	Effect allele	Allele model^a^		Dominant model^b^		Recessive model^b^		Additive model^b^	
			*P*	OR (95% CI)	*P*	OR (95% CI)	*P*	OR (95% CI)	*P*	OR (95% CI)
*SNCA*	rs8180209	A	**.047**	**0.77 (0.60, 0.98)**	.138	0.74 (0.50, 1.10)	.077	0.66 (0.42, 1.05)	**.047**	**0.77 (0.59, 0.99)**
*MCCC1*	rs2270968	G	.091	1.27 (0.99, 1.64)	**.024**	**1.52 (1.06‐2.19)**	.907	0.96 (0.50, 1.84)	.084	1.28 (0.97, 1.71)
*DLG2*	rs7479949	C	.159	0.82 (0.64, 1.06)	.322	0.82 (0.56, 1.21)	**.019**	**0.26 (0.09‐0.80)**	.072	0.73 (0.52, 1.03)
*GBF1*	rs10748818	G	**.043**	**0.72 (0.56, 0.92)**	.093	0.73 (0.50, 1.05)	.089	0.60 (0.33, 1.08)	**<.001** ^c^	**0.37 (0.25‐0.57)**
*MBNL2*	rs4771268	T	**.011**	**0.72 (0.54, 0.96)**	.179	0.77 (0.53, 1.12)	**.003** ^c^	**0.48 (0.29‐0.78)**	**.015**	**0.73 (0.56‐0.94)**

Abbreviations: CI, confidence interval; OR, odds ratio; PD, Parkinson's disease; SNP, single nucleotide polymorphism.

^a^ *P* value, OR and 95% CI were obtained from risk analysis and refer to the risk allele.

^b^ Adjusted for age and gender.

^c^ The statistical significances remained after using Bonferroni correction.

Bold values are indicate the significant results

Since two pairs SNPs of the twelve detected polymorphisms were located on the same chromosome, to explore whether they were in linkage disequilibrium, LD linkage analysis was performed. Linkage patterns were observed between rs1450522 and rs2270968 (*r* ^2^ = .011, *D*′ = 0.177), rs12528068 and rs9261484 (*r* ^2^ = .001, *D*′ = 0.218).

### 3.3 Genotype‐phenotype analysis in LOPD and EOPD

All PD patients were divided into two subgroups: LOPD and EOPD. There were no discrepancies in gender between EOPD and LOPD patients (*P* = .580). For LOPD group, the dominant models of the rs2270968 of *MCCC1* (*P* = .042, OR = 1.48) and the rs9261484 of *TRIM40* (*P* = .039, OR = 1.49) were found to be related to the risk of LOPD (Table 3; Table S4). The rs4771268 of *MBNL2* was found to be associated with PD in both recessive model and the additive model (recessive model: *P* = .007, OR = 0.48; additive model: *P* = .015, OR = 0.72). The rs10748818 of *GBF1* under the additive model remained statistically significant after the Bonferroni correction (*P* < .001, OR = 0.41).

**TABLE 3:** Association of SNPs of candidate genes and odds ratio to LOPD risk

Candidate gene	SNP	Effect allele	Allele model^a^		Dominant model^b^		Recessive model^b^		Additive model^b^	
			*P*	OR (95% CI)	*P*	OR (95% CI)	*P*	OR (95% CI)	*P*	OR (95% CI)
*SNCA*	rs8180209	A	.052	0.77 (0.59‐1.00)	.146	0.74 (0.49, 1.11)	.064	0.94 (0.65, 1.37)	.044	0.76 (0.57, 0.99)
*MCCC1*	rs2270968	G	.152	1.23 (0.93‐1.64)	**.042**	**1.48 (1.01, 2.16)**	.822	0.92 (0.46, 1.85)	.130	1.26 (0.93, 1.10)
*DLG2*	rs7479949	C	.074	0.77 (0.59‐1.03)	.135	0.74 (0.49, 1.10)	.015	0.21 (0.06, 0.74)	.022	0.66 (0.46, 0.94)
*GBF1*	rs10748818	G	.059	0.77 (0.58‐1.01)	.129	0.74 (0.50, 1.09)	.075	0.56 (0.30, 1.06)	**<.001** ^c^	**0.41 (0.27, 0.62)**
*MBNL2*	rs4771268	T	.015	0.71 (0.54‐0.94)	.130	0.74 (0.50, 1.09)	**.007**	**0.48 (0.29, 0.82)**	**.015**	**0.72 (0.55, 0.94)**
*TRIM40*	rs9261484	T	.130	1.26 (0.93‐1.70)	**.039**	**1.49 (1.02, 2.18)**	.926	0.96 (0.43, 2.16)	.118	1.28 (0.94, 1.75)

Abbreviations: CI, confidence interval;LOPD, late‐onset Parkinson's disease; OR, odds ratio; SNP, single nucleotide polymorphism.

^a^ Adjusted for age and gender.

^b^ *P* value, OR and 95% CI were obtained from risk analysis and refer to the risk allele.

^c^ The statistical significance remained after using Bonferroni correction.

Bold values are indicate the significant results

In the analysis between EOPD and HC, the rs10748818 of *GBF1* under the additive model was found to be associated with EOPD after adjustment for age and gender (*P* = .011, OR = 0.20) (Table 4; Table S5). The rs12528068 of *RIMS1* was significantly related to the risk of EOPD under the allele, dominant as well as the additive model with a strong genetic power of 0.871 (allele model: *P* = .034, OR = 0.15; dominant model: *P* = .023, OR = 0.06; additive model: *P* = .024, OR = 0.06). None were statistically significant after the Bonferroni correction.

**TABLE 4:** Association of SNPs of candidate genes and odds ratio to EOPD risk

Candidate gene	SNP	Effect allele	Allele model^a^		Dominant model^b^		Recessive model^b^		Additive model^b^	
			*P*	OR (95% CI)	*P*	OR (95% CI)	*P*	OR (95% CI)	*P*	OR (95% CI)
*SNCA*	rs8180209	A	.441	0.81 (0.47, 1.39)	.383	0.63 (0.22, 1.78)	.633	0.94 (0.35, 2.52)	.411	0.76
*MCCC1*	rs2270968	G	.147	1.46 (0.87, 2.44)	.151	1.71 (0.82, 3.54)	.542	1.25 (0.61, 2.56)	.542	1.25
*DLG2*	rs7479949	C	.590	0.87 (0.53, 1.44)	.064	0.31 (0.09, 1.07)	.588	0.72 (0.07, 7.97)	.822	1.12
*GBF1*	rs10748818	G	.252	0.74 (0.43, 1.24)	.644	0.79 (0.28, 2.18)	.961	0.98 (0.37, 2.58)	**.011**	**0.20 (0.06, 0.70)**
*MBNL2*	rs4771268	T	.244	0.74 (0.44, 1.23)	.828	0.89 (0.32, 2.47)	.178	0.39 (0.10, 1.54)	.373	0.73 (0.37, 1.45)
*RIMS1*	rs12528068	T	**.034**	**0.15 (0.02, 0.53)**	**.023**	**0.06 (0.01, 0.68)**	/	/	**.024**	**0.06 (0.01, 0.70)**

Abbreviations: CI, confidence interval; EOPD, early‐onset Parkinson's disease; OR, odds ratio; SNP, single nucleotide polymorphism.

^a^ Adjusted for age and gender.

^b^ *P* value, OR and 95% CI were obtained from risk analysis and refer to the risk allele.

Bold values are indicate the significant results

### 3.4 Meta‐analysis

**FIGURE 1:**
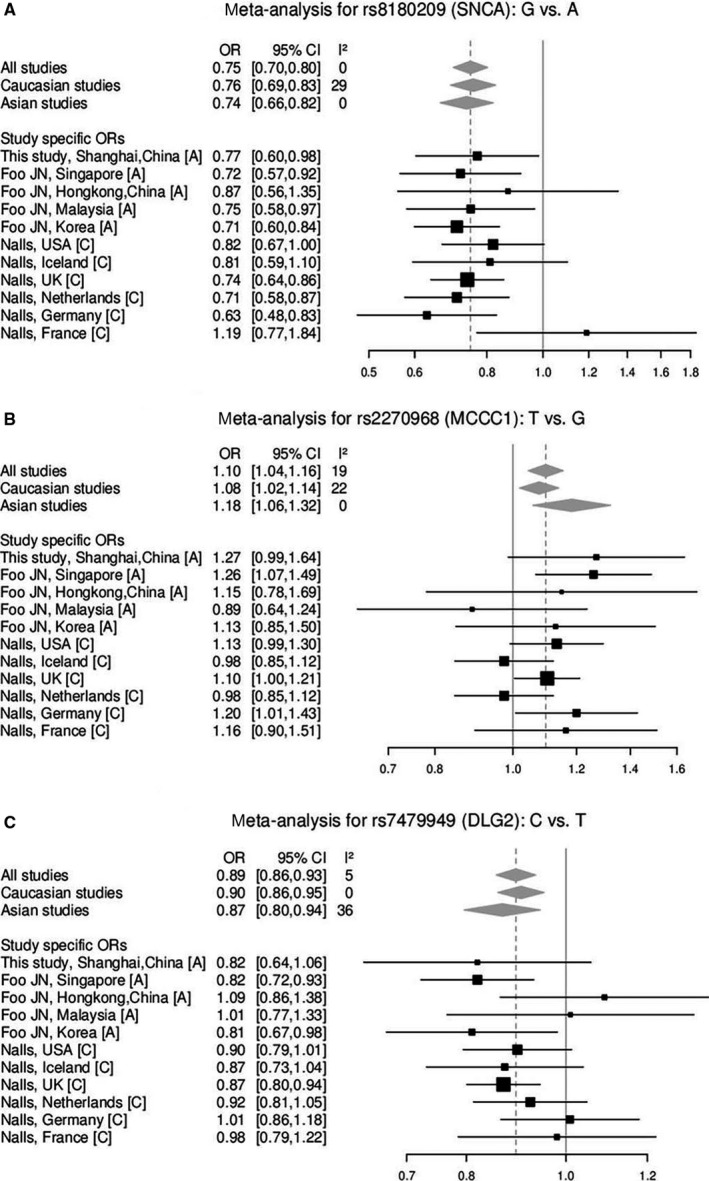
Forest plot of the studies for variants rs8180209, rs2270968 and rs7479949. A, Asian; C, Caucasian

**FIGURE 2:**
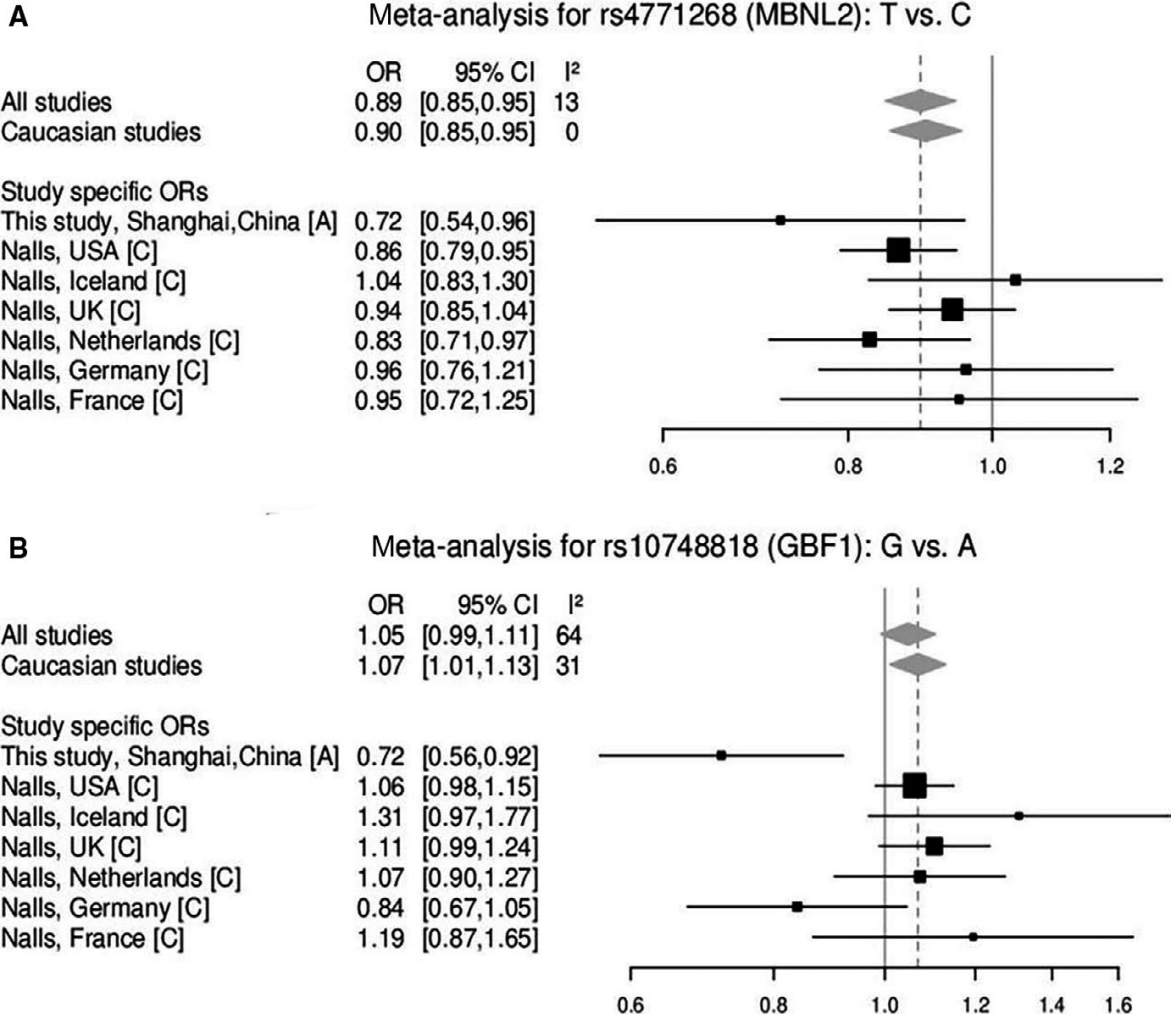
Forest plot of the studies for variants rs10748818 and rs4771268. A, Asian; C, Caucasian

To further verify the results, we performed a meta‐analysis for these loci based on our data and two other available GWAS studies.^10^, ^11^ The meta‐analyses then identified rs8180209 of *SNCA* (*P* < .001, OR = 0.75; Figure 1A) and rs4771268 of *MBNL2* (*P* < .001, OR = 0.90; Figure 2B), which are in accordance with the results from our cohort. Substantial heterogeneity was found in rs10748818 of *GBF1* in these studies (*I* ^2^ = 64%, *P* = .092; Figure 2A) mainly attributed to different ORs in Asian population in our study. But rs2270968 of *MCCC1* (*P* < .001, OR = 1.10; Figure 1B) and rs7479949 of *DLG2* (*P* < .001, OR = 0.89; Figure 1C) which were not significantly associated with PD in allele model in our cohort still turned to be significant alleles for PD following the meta‐analysis. However, the association was in the same direction as in the reference studies with the magnitude of risk similar or greater than previously reported for rs2270968 and rs7479949.

### 3.5 Functional prediction and validation

To fully understand the influence of relevant loci on the onset of PD, we selected 7 candidate loci based on the above results to explore the association between the genotype and the gene expression by performing eQTL analysis using GTEx and Braineac data sets. Except for the rs9261484 of *TRIM40*, all the other 6 SNPs were shown to alter the relevant gene expression in the specific brain regions (Figure S1; Table S6). The A allele of rs8180209 decreased *SNCA* expression in white matter (*P* = .017) (Figure 1A). The G allele of rs2270968 increased the *MCCC1* expression in various brain regions, including frontal cortex (*P* = .038), hippocampus (*P* = .010), putamen (*P* = .007), substantia nigra (*P* = .003) and thalamus (*P* = .042) (Figure 1B). The C allele of rs7479949 elevated the *DLG2* expression in the occipital cortex (*P* = .038) and the temporal cortex (*P* = .048) (Figure 1C). The G allele of rs10748818 increased the *GBF1* in the frontal cortex (*P* = .026) (Figure 1D). The T allele of rs4771268 attenuated the *MBNL2* expression in white matter (*P* = .009) (Figure 1D). And finally, the T allele of rs12528068 increased the *RIMS1* expression in the hippocampus (*P* = .044) (Figure 1E).

Moreover, in the GTEx database, sQTLs (splicing QTLs) showed that the rs8180209 of *SNCA* altered the splicing or alternative splicing of the intron to modulate the expression of *SNCA* in the cortex and nerve (Figure S2; Table S7). And the eQTLs of GTEx results were also in agreement with the Braineac analysis that the G allele of rs2270968 enhanced the *MCCC1* expression in various brain regions (Figure S3; Table S7).

The rs10748818 of *GBF1* was the only locus of five SNPs located on the genetic promoter region. Hence, we performed dual‐luciferase reporter gene assay to test whether this variation altered *GBF1* promoter transcriptional activity. However, allele alteration of rs10748818 had no effect on *GBF1* promoter transcriptional activity in Jurkat cells and SH‐SY5Y (Figure S4).

## 4 DISCUSSION

In our study, we replicated that the following SNPs were associated with PD: the rs8180209 of *SNCA* (allele model, additive model), the rs2270968 of *MCCC1* (dominant model), the rs7479949 of *DLG2* (recessive model), the rs10748818 of *GBF1* (allele model and additive model) and the rs4771268 of *MBNL2* (allele model, recessive model and additive model). To the best of our knowledge, our study is the first to show the association of SNPs in *GBF1* and *MBNL2* genes with PD, and to replicate the associations of other SNPs (in *SNCA*, *MCCC1* and *DLG2* genes) with PD in the southern Chinese population. Our study, however, failed to replicate the association of the reported SNPs with PD in Caucasians, in southern Chinese PD patients. The differences in allele frequency and distributions, due to ethnic diversity, might partially explain the results.

*SNCA* (synuclein alpha) plays an important role in PD pathogenesis, by encoding two different isoforms of α‐synuclein, which is considered as a major component of Lewy bodies and a hallmark of PD.^19^, ^20^ A plethora of evidence suggests that the genetic variability in the *SNCA* gene is associated with an increased risk of PD, which is true not only for the rare Mendelian forms but also for the common sporadic forms of PD.^21^ Many case‐control studies and meta‐analyses have been conducted to study the relationship between the *SNCA* polymorphisms and susceptibility to PD. The *SNCA* variants rs356219,^22^ rs356182^23^ and rs2736990^24^ have been speculated to be associated with the increased risk of PD in the Han Chinese populations. A few studies have reported the association of PD with the *SNCA* variant rs8180209, but interestingly in our eQTLs studies, the minor A allele played a protective role by decreasing the *SNCA* expression, probably by altering the splicing isoforms. Large‐scale and better‐designed case‐control studies on populations of different ethnicities are required to thoroughly investigate the role of rs8180209 in the pathogenesis of PD.

*MCCC1* (methylcrotonyl‐coenzyme A carboxylase 1) encodes for the α subunit of MCC, a biotin‐dependent mitochondrial enzyme, which plays a crucial role in leucine catabolism.^25^ The studies on the association of PD and *MCCC1* have mainly focused on genetic variants and susceptibility to PD. Recent studies showed that the SNPs of *MCCC1* showed association with age at onset of PD and motor progression.^26^, ^27^ Several case‐control studies confirm that in the Chinese Han population, rs11711441 variant of *MCCC1* is associated with a lower risk of PD,^28^ while rs12637471 variant is associated with an increased risk.^29^ Moreover, our study showed that *MCCC1* variant rs2270968 is associated with an increased risk of PD as well as LOPD as indicated by the elevated gene expression. Understanding the details of the molecular basis of this association would be of considerable importance.

*DLG2* (Discs Large MAGUK Scaffold Protein 2) encodes for the discs‐large membrane‐associated guanylate kinase scaffolding protein 2, a member of the membrane‐associated guanylate kinase (MAGUK) family. The *DLG2* variant rs3793947 polymorphism AA genotype has been shown to be significantly associated with a protective effect for PD in Caucasian and Taiwanese populations.^30^ The *DLG2* encoded protein has been shown to interact with the glutamate receptors,^31^ it has been shown to be involved in the Fyn‐dependent tyrosine phosphorylation of NR2 subunits of N‐methyl‐D‐aspartate glutamate receptors,^32^ and the excitotoxicity mediated by these receptors has been reported to be involved in the pathogenesis of PD.^33^ It has been speculated that the loss of dopamine (DA) caused by striatal DA impairment in PD may lead to an uninhibited glutamate‐induced calcium signalling and subsequent cell death.^34^ Therefore, the functional and the cell death pathway regulation by *DLG2* variant rs7479949 requires further investigation.

*GBF1* (Golgi Brefeldin A Resistant Guanine Nucleotide Exchange Factor 1) encodes for the Golgi‐specific Brefeldin A resistance factor 1, an enzyme which selectively modulates the ER‐Golgi trafficking.^35^ The rs3758549 locus is localized in the promoter region of both *GBF1* and *PITX3*. For *PITX3*, rs3758549 is reported to be significantly associated with the risk of PD in the Asian population.^36^ But for *GBF1*, its association with PD has not been studied in detail. *GBF1* is shown to modulate the rate of anterograde trafficking to control protein secretion and its carrier organelle,^37^ the axonal anterograde transport was impaired in the MPTP model, and anterograde axonal transport of glial cell line‐derived neurotrophic factor (GDNF) was shown to be adversely affected in the 6‐OHDA model.^38^ Accumulation of misfolded/unfolded α‐synuclein in the endoplasmic reticulum (ER) and disruptions in protein clearance mechanisms causes activation of ER stress mechanisms which could be observed in post‐mortem tissue from sporadic human PD brains and in many animal models of PD.^39^ *GBF1* is also shown to modulate the ER‐Golgi response to the external environment.^40^ All these studies suggest association of *GBF1* with PD, but more detailed investigation is required. Additionally, the rs10748818 failed to affect *GBF1* promoter transcriptional activity in human T lymphocyte cells and human neuroblastoma cells. The clinical and genetic heterogeneity can be explained, at least partly, by tissue‐specific expression pattern and the impact of environmental factors.

*MBNL2* (muscleblind‐like splicing regulator 2) encodes for the muscleblind‐like protein 2, which belongs to a conserved family of RNA‐binding proteins. The association of *MBNL2* and the myotonic dystrophy has been reported in the literature where the *MBNL2* knockout mice were able to recapitulate cardinal features of myotonic dystrophy.^41^ More studies, however, are required to understand the association between the *MBNL2* gene and PD.

There were certain limitations in our study which cannot be understated. Firstly, the genetic powers of most of the SNPs under study were low, because of the small sample size. As the samples of EOPD patients were limited, marginally significant associations should be interpreted cautiously. Secondly, being a single‐centred study, larger sized and multi‐centred cohort studies are needed to further validate the result. Lastly, in this study, all new loci recently identified by the GWAS were not included. The primary reason was that the minor allele frequencies of these SNPs were rare or close to another allele.

In conclusion, in our study the rs8180209 variant of *SNCA*, the rs2270968 variant of *MCCC1*, the rs7479949 variant of *DLG2*, the rs10748818 variant of *GBF1* and the rs4771268 variant of *MBNL2* were found to be associated with the susceptibility to PD in the southern Chinese population. Additionally, the rs9261484 variant of *TRIM40* and the rs12528068 variant of *RIMS1* were found to be associated with LOPD and EOPD, respectively. Further exploration of the genetic risk factors for PD in the Asian populations would require larger and more diverse PD cohorts.

## CONFLICT OF INTEREST

None.

## AUTHOR CONTRIBUTIONS

JL designed the study, provided financial support and revised the manuscript. WK revised the manuscript. AZ, YL, MN, GL, NL and LZ collected the data. AZ and YL carried out the genetic analyses and performed data analysis. AZ wrote the manuscript. All the co‐authors contributed to revising the manuscript for intellectual content and approved the final version for publication.

## Supporting information

Fig S1

Fig S2

Fig S3

Fig S4

Table S1‐S7

## Data Availability

All data relevant to the study are included in the article or uploaded as supplementary information.
