# Supplementary figures and images for: SNPs in SNCA, MCCC1, DLG2, GBF1 and MBNL2 are associated with Parkinson's disease in southern Chinese population
Source: J Cell Mol Med. 2020 Jul 11;24(15):8744–52. doi: 10.1111/jcmm.15508 (PMC7412680; doi:10.1111/jcmm.15508)

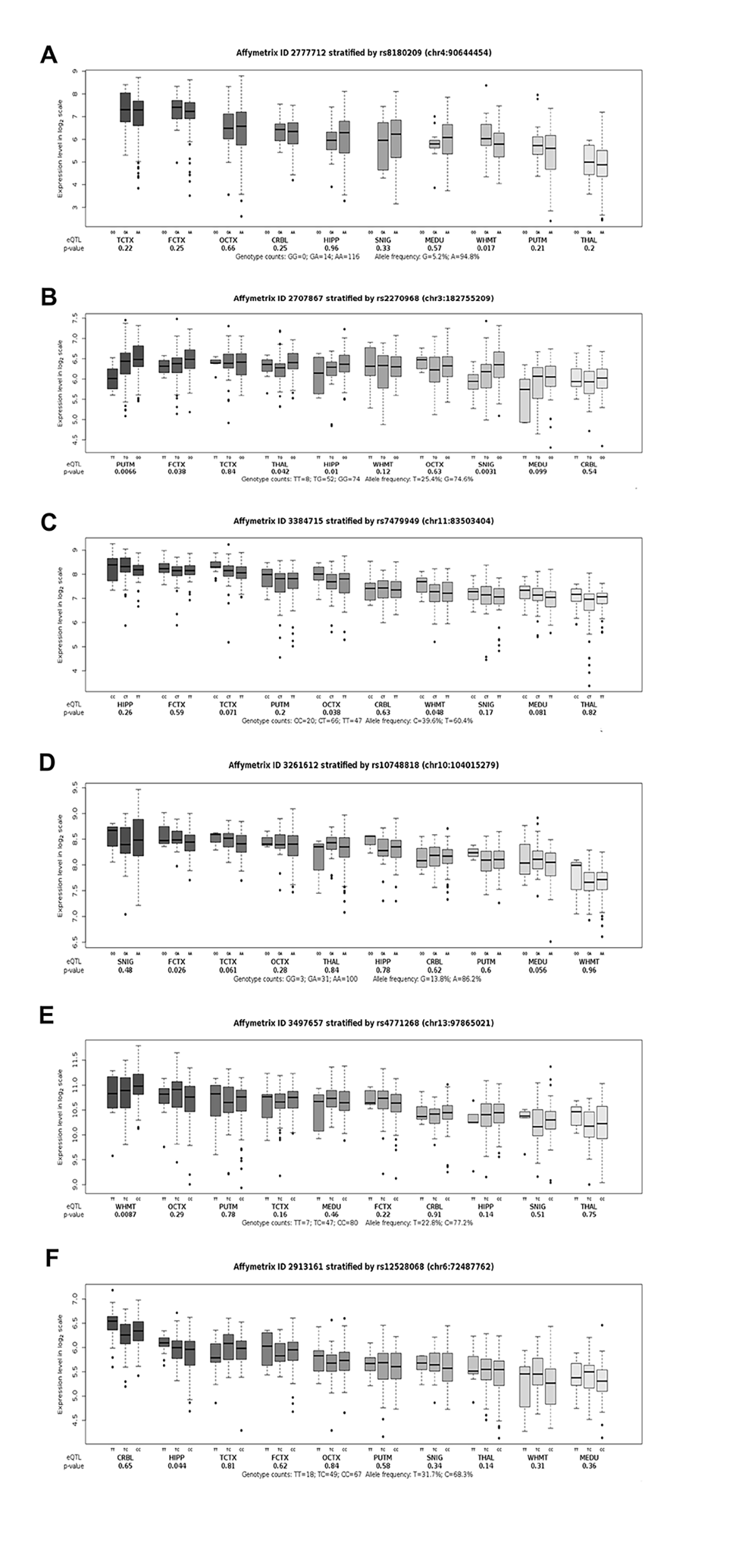

Supplement: Supplementary file 1 — Fig S1 [file JCMM-24-8744-s001.tif]

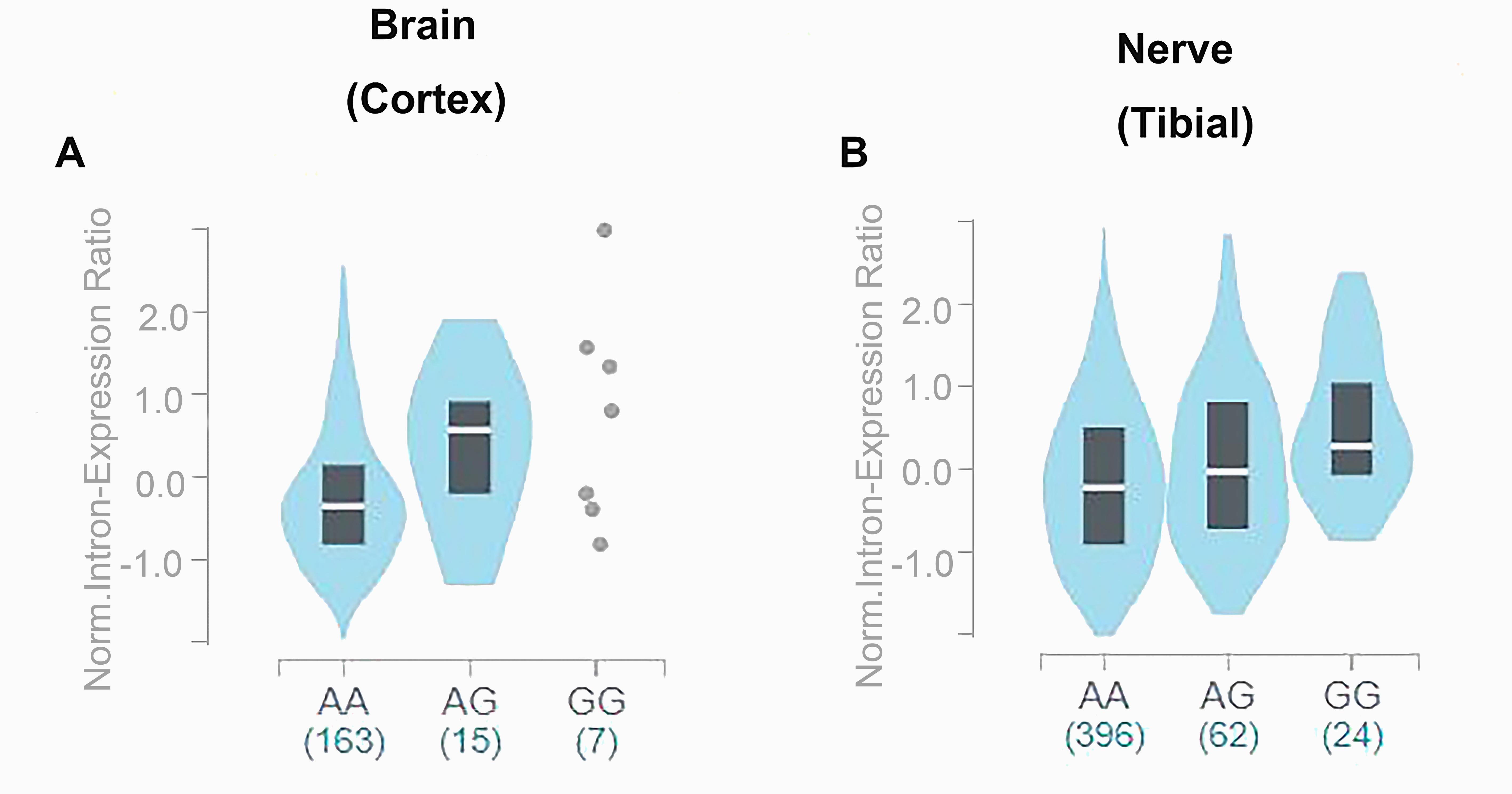

Supplement: Supplementary file 2 — Fig S2 [file JCMM-24-8744-s002.jpg]

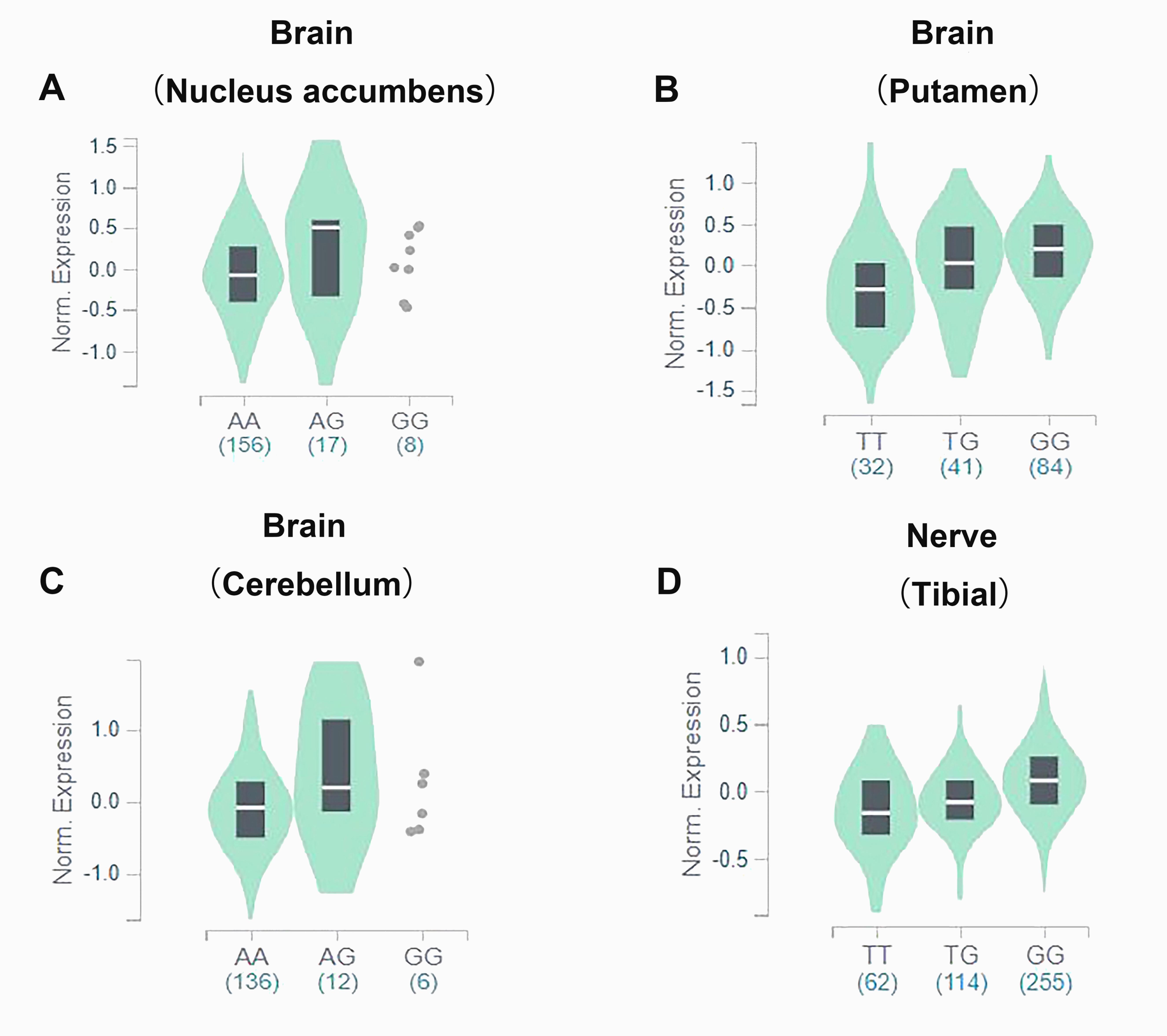

Supplement: Supplementary file 3 — Fig S3 [file JCMM-24-8744-s003.jpg]

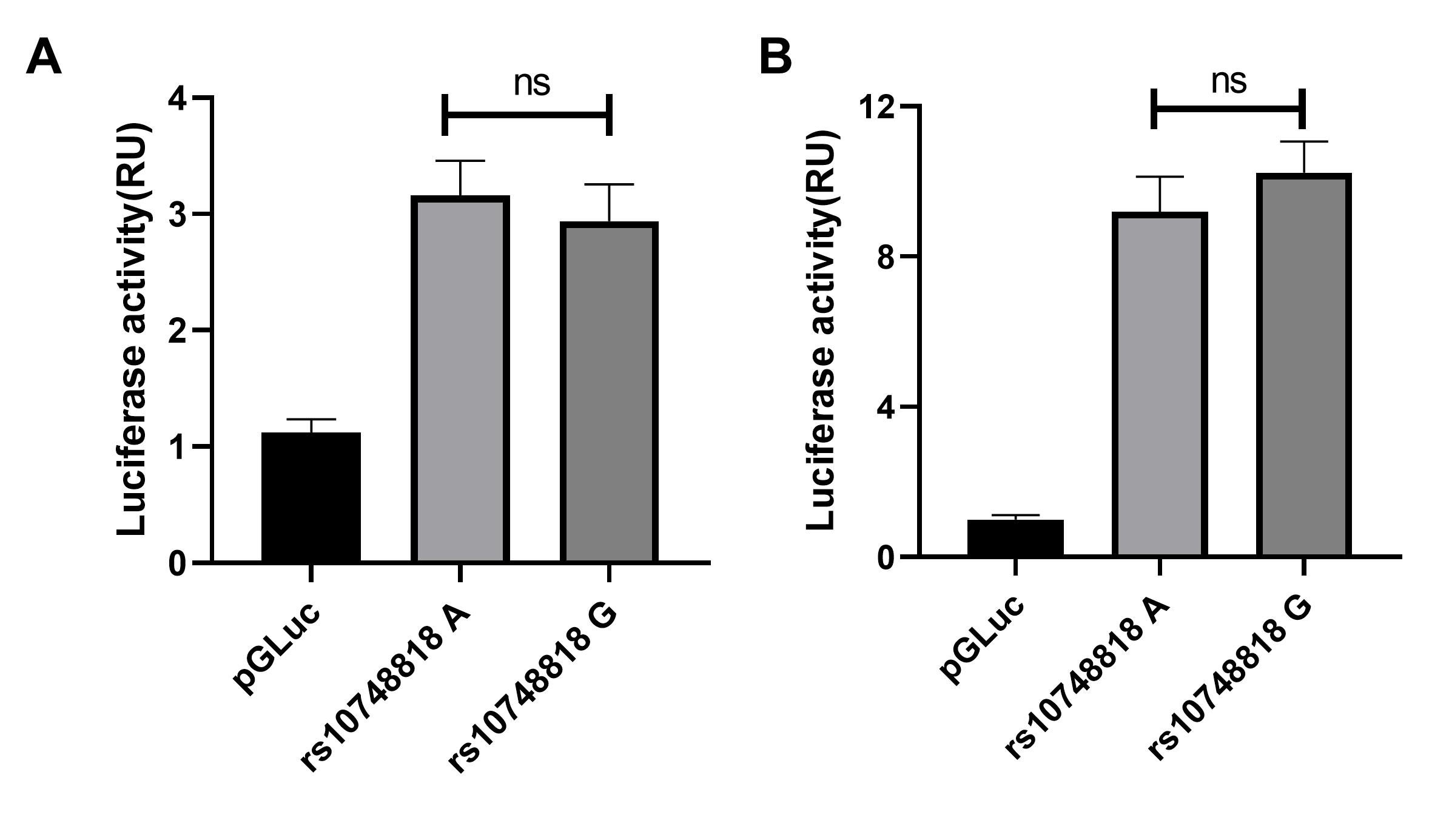

Supplement: Supplementary file 4 — Fig S4 [file JCMM-24-8744-s004.jpg]
