# Supplementary material for: SNPs in SNCA, MCCC1, DLG2, GBF1 and MBNL2 are associated with Parkinson's disease in southern Chinese population
Source: J Cell Mol Med. 2020 Jul 11;24(15):8744–52. doi: 10.1111/jcmm.15508 (PMC7412680; doi:10.1111/jcmm.15508)
Supplement: Supplementary file 5 — Table S1‐S7 [file JCMM-24-8744-s005.doc]

Table S1. Primers of tested single nucleotide polymorphisms

| SNPs | chr | Forward Primers | Reverse Primers | Elongation Primers | Reference |
| --- | --- | --- | --- | --- | --- |
| rs8180209 | 4 | TCCGGTTCATTTCTGACACCA | CCTGTGGGTCAATGCTAACTCC | CACAAAGAATGGGAGAATTGCT | Foo 2017  Nalls 2018 |
| rs2270968 | 3 | TATTGTTTTCCCCACTCAGCAGG | CTGTTTGTGGTGTTGAGGGATGAA | CTCTTCCTAGATTGTTGGACTGC | Foo 2017  Nalls 2018 |
| rs7479949 | 11 | TGGAAAGACTTCTTGGTGAAAGG | TTAATTGTATGTCTTTTCATTGTGCC | AGAAAACAAAAACAGGCCATAAT | Foo 2017  Nalls 2018 |
| rs10748818 | 10 | TTAGACCTTTGCTGTCTTAGAAAGTGG | CTGGATGACTATGGAACTGAGTGG | GAGGAGTGAAAGGAAGACTAACTTT | Nalls 2018 |
| rs61169879 | 17 | ACATAACCAAAAGGAGAAATCAATCA | TGTTGAAGATGTTTATGGCTGAAATC | AGTCTAAACCGCTTGACCATAA | Nalls 2018 |
| rs9261484 | 6 | GCAACACCAAGAGTTTCATTTAAGA | AATATTGTCTTTGYCTGGATGGA | GAGACACATCAAAATAGGATCTTACATA | Nalls 2018 |
| rs4771268 | 13 | TAGTGTCATGGATTTCACACAATAGC | AATGTGTTACTCATCTCTTTCCTCCA | CTATGTACATATTTAATAAATAGATGTCTCTC | Nalls 2018 |
| rs11610045 | 12 | AGCCATTCCCTCCACTTCCG | CCCACACTCCCACTGACTTCTTG | ATCTGCCCTCTCTTGCCC | Nalls 2018 |
| rs2248244 | 21 | GCCATAGCTGTTTTTCATTAATCG | TCCCTCTTCTGTAACTTGCATGAAT | TAGGTTTAAAAACTGGGCAT | Nalls 2018 |
| rs12528068 | 6 | GCAGCATCATTTTCCTTCTGC | ATACAAGAGGCTGAACAAATCCTAAG | TGTTTTTCCACTATCTTACCAGC | Nalls 2018 |
| rs2904880 | 16 | GCCAAAGACCGCCCTGAGAT | TCCCCAGTCATCACCATACCCT | GAGGGAGAGCCTCCGTGT | Nalls 2018 |
| rs1450522 | 3 | ACCCAGAGAGGAGAACTAACCCA | TTCTCAAAGCAAGTCTCCGTGATA | GTTCTTCAGCCTTGCTCCTTC | Nalls 2018 |

SNP = single nucleotide polymorphism

**Table S2. Association of SNPs of candidate genes and odds ratio to PD risk**

| Candidate Gene | SNP | HWE *p* value | MAF (case/control) | Allele | | | | Dominant Model | | |
| --- | --- | --- | --- | --- | --- | --- | --- | --- | --- | --- |
|  |  |  |  | Effect allele | *p^a^* | OR^a^ | 95%CI^a^ | *p* | OR | 95%CI |
| *SNCA* | rs8180209 | 0.839 | 0.42/0.48 | **A** | **0.047** | **0.77** | **0.60-0.99** | 0.126 | 0.74 | (0.50,1.09) |
| *MCCC1* | rs2270968 | 0.973 | 0.34/0.29 | G | 0.091 | 1.27 | 0.99-1.67 | 0.023 | 1.52 | (1.06, 2.09) |
| *DLG2* | rs7479949 | 0.452 | 0.34/0.38 | C | 0.159 | 0.82 | 0.64-1.08 | 0.341 | 0.83 | (0.56, 1.22) |
| *GBF1* | rs10748818 | 0.202 | 0.33/0.39 | **G** | **0.043** | **0.76** | **0.58-0.99** | 0.092 | 0.73 | (0.50, 1.05) |
| *BRIP1* | rs61169879 | 0.627 | 0.42/0.39 | C | 0.336 | 1.13 | 0.88-1.47 | 0.418 | 1.16 | (0.80, 1.70) |
| *TRIM40* | rs9261484 | 0.948 | 0.28/0.25 | T | 0.227 | 0.84 | 0.63-1.12 | 0.111 | 0.75 | (0.52, 1.07) |
| *MBNL2* | rs4771268 | 0.200 | 0.37/0.45 | **T** | **0.011** | **0.72** | **0.55-0.93** | 0.169 | 0.77 | (0.53, 1.12) |
| *FBRSL1* | rs11610045 | 0.759 | 0.12/0.12 | A | 0.939 | 0.98 | 0.66-1.46 | 0.641 | 0.90 | (0.58, 1.40) |
| *DYRK1A* | rs2248244 | 0.390 | 0.37/0.35 | A | 0.498 | 1.10 | 0.84-1.42 | 0.946 | 1.01 | (0.70, 1.46) |
| *RIMS1* | rs12528068 | 0.313 | 0.06/0.08 | T | 0.202 | 1.37 | 0.84-2.25 | 0.264 | 1.35 | (0.80, 2.27) |
| *CD19* | rs2904880 | 0.338 | 0.12/0.10 | C | 0.473 | 1.16 | 0.77~1.74 | 0.326 | 1.25 | (0.80, 1.94) |
| *SPTSSB* | rs1450522 | 0.339 | 0.40/0.43 | A | 0.334 | 0.88 | 0.68-1.14 | 0.339 | 0.83 | (0.57, 1.22) |

| Candidate Gene | SNP | Genetic Power | Dominant Model (adjusted)^b^ | | | | Recessive Model | | | Recessive Model (adjusted)^b^ | | |
| --- | --- | --- | --- | --- | --- | --- | --- | --- | --- | --- | --- | --- |
|  |  |  | *p* | OR | 95%CI | | *p* | OR | 95%CI | *p* | OR | 95%CI |
| *SNCA* | rs8180209 | 0.086 | 0.138 | 0.74 | (0.50, 1.10) | | 0.082 | 0.67 | (0.43,1.05) | 0.077 | 0.66 | (0.42, 1.05) |
| *MCCC1* | rs2270968 | 0.107 | **0.024** | **1.52** | **(1.06, 2.19)** | | 0.934 | 0.97 | (0.51,1.86) | 0.907 | 0.96 | (0.50, 1.84) |
| *DLG2* | rs7479949 | 0.119 | 0.322 | 0.82 | (0.56, 1.21) | | 0.016 | 0.26 | (0.09,0.78) | **0.019** | **0.26** | **(0.09,0.80)** |
| *GBF1* | rs10748818 | 0.087 | 0.093 | 0.73 | (0.50, 1.05) | | 0.079 | 0.59 | (0.32, 1.06) | 0.089 | 0.60 | (0.33, 1.08) |
| *BRIP1* | rs61169879 | 0.098 | 0.429 | 1.16 | (0.80, 1.69) | | 0.461 | 1.19 | (0.74, 1.92) | 0.524 | 1.17 | (0.73, 1.88) |
| *TRIM40* | rs9261484 | 0.121 | 0.109 | 0.75 | (0.52, 1.07) | | 0.853 | 0.93 | (0.29, 2.00) | 0.819 | 0.91 | (0.43, 1.97) |
| *MBNL2* | rs4771268 | 0.062 | 0.179 | 0.77 | (0.53, 1.12) | | 0.003 | 0.48 | (0.72, 0.78) | **0.003**^c^ | **0.48** | **(0.29, 0.78)** |
| *FBRSL1* | rs11610045 | 0.050 | 0.668 | 0.91 | (0.70, 1.41) | | 0.305 | 0.52 | (0.15, 1.81) | 0.264 | 0.49 | (0.14, 1.71) |
| *DYRK1A* | rs2248244 | 0.082 | 0.975 | 1.01 | (0.70, 1.45) | | 0.207 | 0.71 | (0.42, 1.21) | 0.207 | 0.71 | (0.41, 1.21) |
| *RIMS1* | rs12528068 | 0.304 | 0.278 | 1.34 | (0.79, 2.26) | | 0.373 | 2.81 | (0.29, 27.16) | 0.973 | 2.99 | (0.31, 29.02) |
| *CD19* | rs2904880 | 0.128 | 0.365 | 1.23 | (0.79, 1.92) | | 0.473 | 1.87 | (0.34,10.30) | 0.448 | 1.94 | (0.35, 10.77) |
| *SPTSSB* | rs1450522 | 0.100 | 0.332 | 0.83 | (0.56, 1.21) | | 0.540 | 0.86 | (0.53,1.39) | 0.595 | 0.88 | (0.54, 1.42) |
| Candidate Gene | SNP | Additive model | | | | Additive model (adjusted)^b^ | | | | Number of samples tested | | |
|  |  | *p* | OR | 95%CI | | *p* | OR | 95%CI | | Case | | Control |
| *SNCA* | rs8180209 | 0.045 | 0.77 | (0.59,0.99) | | **0.047** | **0.77** | **(0.59,0.99)** | | 231 | | 249 |
| *MCCC1* | rs2270968 | 0.079 | 1.29 | (0.97, 1.71) | | 0.084 | 1.28 | (0.97, 1.71) | | 231 | | 249 |
| *DLG2* | rs7479949 | 0.073 | 0.73 | (0.52, 1.03) | | 0.072 | 0.73 | (0.52, 1.03) | | 231 | | 249 |
| *GBF1* | rs10748818 | 0.000 | 0.38 | (0.25, 0.57) | | **0.000**^c^ | **0.37** | **(0.25, 0.57)** | | 231 | | 249 |
| *BRIP1* | rs61169879 | 0.344 | 1.13 | (0.88, 1.46) | | 0.379 | 1.12 | (0.87, 1.45) | | 231 | | 249 |
| *TRIM40* | rs9261484 | 0.214 | 1.21 | (0.90, 1.62) | | 0.218 | 1.21 | (0.90, 1.62) | | 231 | | 249 |
| *MBNL2* | rs4771268 | 0.014 | 0.72 | (0.56, 0.94) | | **0.015** | **0.73** | **(0.56, 0.94)** | | 231 | | 249 |
| *FBRSL1* | rs11610045 | 0.942 | 0.99 | (0.68, 1.44) | | 0.990 | 1.00 | (0.70, 1.43) | | 231 | | 249 |
| *DYRK1A* | rs2248244 | 0.498 | 1.10 | (0.84, 1.43) | | 0.513 | 1.09 | (0.84, 1.43) | | 231 | | 249 |
| *RIMS1* | rs12528068 | 0.213 | 0.74 | (0.45, 1.19) | | 0.218 | 0.74 | (0.46, 1.20) | | 231 | | 249 |
| *CD19* | rs2904880 | 0.474 | 1.16 | (0.77, 1.74) | | 0.525 | 1.14 | (0.76, 1.71) | | 231 | | 249 |
| *SPTSSB* | rs1450522 | 0.322 | 0.88 | (0.67, 1.14) | | 0.339 | 0.88 | (0.68, 1.15) | | 231 | | 249 |
| CI= confidence interval; HWE = Hardy-Weinberg Equilibrium; MAF = minor allele frequency; OR = odds ratio; PD = Parkinson’s Disease; SNP = single nucleotide polymorphism.  ^a^*p* value, OR and 95% CI were obtained from risk analysis and refer to the risk allele.  ^b^ Adjusted for age and gender.  ^c^The statistical significances remained after using Bonferroni correction.  Bold character showed p<0.05 in the indicated model. | | | | | | | | | | | | |

**Table S3. Association of SNPs of candidate genes and odds ratio to PD risk validated in another independent cohort**.

| Candidate Gene | SNP | Effect allele | Allele model ^a^ | |  | Dominant model ^b^ | |  | Recessive model ^b^ | |  | Additive model ^b^ | |
| --- | --- | --- | --- | --- | --- | --- | --- | --- | --- | --- | --- | --- | --- |
|  |  |  | *p* | OR (95%CI) |  | *p* | OR (95%CI) |  | *p* | OR (95%CI) |  | *p* | OR (95%CI) |
| ***SNCA*** | rs8180209 | A | **0.042** | **0.79**  **(0.62, 0.94)** |  | 0.251 | 0.88  (0.47, 1.24) |  | 0.126 | 0.97  (0.73, 1.29) |  | **0.038** | **0.74**  **(0.58,0.97)** |
| ***MCCC1*** | rs2270968 | G | 0.086 | 1.13  (0.92, 1.42) |  | **0.030** | **1.39**  **(1.05-1.87)** |  | 0.405 | 0.62  (0.41, 1.16) |  | 0.091 | 1.19  (0.72, 1.96) |
| ***DLG2*** | rs7479949 | C | 0.247 | 1.15  (0.63, 1.37) |  | 0.462 | 0.96  (0.67, 1.13) |  | **0.036** | **0.58**  **(0.04-0.77)** |  | 0.064 | 0.69  (0.59, 1.01) |
| ***GBF1*** | rs10748818 | G | **0.045** | **0.65**  **(0.45, 0.89)** |  | 0.056 | 0.94  (0.31, 1.26) |  | 0.314 | 0.79  (0.26, 1.03) |  | **<0.001**^c^ | **0.51**  **(0.33-0.62)** |
| ***MBNL2*** | rs4771268 | T | **0.028** | **0.58**  **(0.39, 0.78)** |  | 0.117 | 0.97  (0.61, 1.17) |  | **0.020** | **0.64**  **(0.18-0.84)** |  | 0.119 | 0.63  (0.51-1.15) |

CI: Confidence Interval, OR: odd ratio, PD: Parkinson’s Disease; SNP: single nucleotide polymorphism, ^a^*p* value, OR and 95% CI were obtained from risk analysis and refer to the risk allele, ^b^ Adjusted for age and gender, ^c^ The statistical significances remained after using Bonferroni correction.

**Table S4**. Association of SNPs of candidate genes and odds ratio to LOPD risk

| Candidate Gene | SNP | HWE *p* value | MAF (case/control) | Allele | | | | | | Dominant Model | | | |
| --- | --- | --- | --- | --- | --- | --- | --- | --- | --- | --- | --- | --- | --- |
|  |  |  |  | Effect allele | *p^a^* | | OR^a^ | | 95%CI^a^ | *p* | OR | | 95%CI |
| *SNCA* | rs8180209 | 0.839 | 0.42/0.49 | A | 0.052 | | 0.77 | | 0.59-1.00 | 0.160 | 0.75 | | (0.49, 1.12) |
| *MCCC1* | rs2270968 | 0.973 | 0.34/0.29 | G | 0.152 | | 1.23 | | 0.93-1.64 | 0.041 | 1.49 | | (1.02, 2.18) |
| *DLG2* | rs7479949 | 0.452 | 0.33/0.38 | C | 0.074 | | 0.77 | | 0.59-1.03 | 0.136 | 0.74 | | (0.50, 1.10) |
| *GBF1* | rs10748818 | 0.202 | 0.33/0.39 | G | 0.059 | | 0.77 | | 0.58-1.01 | 0.121 | 0.74 | | (0.50, 1.08) |
| *BRIP1* | rs61169879 | 0.627 | 0.42/0.39 | C | 0.426 | | 1.11 | | 0.85-1.46 | 0.461 | 1.16 | | (0.78, 1.71) |
| *TRIM40* | rs9261484 | 0.948 | 0.29/0.25 | T | 0.130 | | 1.26 | | 0.93-1.70 | 0.048 | 1.46 | | (1.00, 2.13) |
| *MBNL2* | rs4771268 | 0.200 | 0.37/0.45 | T | 0.015 | | 0.71 | | 0.54-0.94 | 0.153 | 0.75 | | (0.51, 1.11) |
| *FBRSL1* | rs11610045 | 0.759 | 0.13/0.12 | A | 0.661 | | 1.09 | | 0.73-1.64 | 0.993 | 1.00 | | (0.64, 1.57) |
| *DYRK1A* | rs2248244 | 0.390 | 0.37/0.35 | A | 0.519 | | 1.09 | | 0.83-1.44 | 0.992 | 1.00 | | (0.68, 1.47) |
| *RIMS1* | rs12528068 | 0.313 | 0.07/0.08 | T | 0.491 | | 1.19 | | 0.72-1.96 | 0.594 | 0.87 | | (0.51, 1.47) |
| *CD19* | rs2904880 | 0.338 | 0.12/0.10 | C | 0.527 | | 1.14 | | 0.75-1.75 | 0.409 | 1.21 | | (0.76, 1.93) |
| *SPTSSB* | rs1450522 | 0.339 | 0.42/0.44 | A | 0.606 | | 0.93 | | 0.71-1.22 | 0.621 | 0.90 | | (0.60, 1.35) |
| Candidate Gene | SNP | Genetic Power | Dominant Model (adjusted)^b^ | | | | Recessive Model | | | Recessive Model (adjusted)^b^ | | | |
|  |  |  | *p* | OR | 95%CI | | *p* | OR | 95%CI | *p* | OR | | 95%CI |
| *SNCA* | rs8180209 | 0.091 | 0.146 | 0.74 | (0.49, 1.11) | | 0.060 | 0.63 | (0.39, 1.02) | 0.064 | 0.94 | | (0.65, 1.37) |
| *MCCC1* | rs2270968 | 0.144 | **0.042** | **1.48** | **(1.01, 2.16)** | | 0.776 | 0.91 | (0.45, 1.81) | 0.822 | 0.92 | | (0.46, 1.85) |
| *DLG2* | rs7479949 | 0.109 | 0.135 | 0.74 | (0.49, 1.10) | | 0.020 | 0.23 | (0.07, 0.79) | 0.015 | 0.21 | | (0.06, 0.74) |
| *GBF1* | rs10748818 | 0.097 | 0.129 | 0.74 | (0.50, 1.09) | | 0.095 | 0.59 | (0.31, 1.10) | 0.075 | 0.56 | | (0.30, 1.06) |
| *BRIP1* | rs61169879 | 0.091 | 0.442 | 1.17 | (0.79, 1.73) | | 0.600 | 1.14 | (0.70, 1.88) | 0.475 | 1.20 | | (0.73, 1.99) |
| *TRIM40* | rs9261484 | 0.155 | **0.039** | **1.49** | **(1.02, 2.18)** | | 0.865 | 0.93 | (0.42, 2.08) | 0.926 | 0.96 | | (0.43, 2.16) |
| *MBNL2* | rs4771268 | 0.067 | 0.130 | 0.74 | (0.50, 1.09) | | 0.006 | 0.48 | (0.29, 0.81) | **0.007** | **0.48** | | **(0.29, 0.82)** |
| *FBRSL1* | rs11610045 | 0.074 | 0.996 | 1.00 | (0.63, 1.57) | | 0.194 | 2.28 | (0.66, 7.91) | 0.232 | 2.15 | | (0.61, 7.50) |
| *DYRK1A* | rs2248244 | 0.078 | 0.929 | 1.02 | (0.69, 1.50) | | 0.201 | 1.44 | (0.83, 2.50) | 0.180 | 1.47 | | (0.84, 2.56) |
| *RIMS1* | rs12528068 | 0.167 | 0.622 | 0.87 | (0.51, 1.49) | | 0.457 | 0.42 | (0.04, 4.10) | 0.543 | 0.49 | | (0.05, 4.81) |
| *CD19* | rs2904880 | 0.115 | 0.338 | 1.26 | (0.79, 2.01) | | 0.602 | 0.64 | (0.12, 3.50) | 0.690 | 0.71 | | (0.13, 3.94) |
| *SPTSSB* | rs1450522 | 0.069 | 0.711 | 0.93 | (0.62, 1.39) | | 0.726 | 0.92 | (0.56, 1.51) | 0.635 | 0.89 | | (0.54, 1.46) |
| Candidate Gene | SNP | Additive model | | | | Additive model (adjusted)^b^ | | | | Number of sample tested | | | |
|  |  | *p* | OR | 95%CI | | *p* | OR | 95%CI | | Case | | Control | |
| *SNCA* | rs8180209 | 0.045 | 0.76 | (0.58, 0.99) | | 0.044 | 0.76 | (0.57, 0.99) | | 195 | | 249 | |
| *MCCC1* | rs2270968 | 0.139 | 1.25 | (0.93, 1.69) | | 0.130 | 1.26 | (0.93, 1.10) | | 195 | | 249 | |
| *DLG2* | rs7479949 | 0.025 | 0.67 | (0.47, 0.95) | | 0.022 | 0.66 | (0.46, 0.94) | | 195 | | 249 | |
| *GBF1* | rs10748818 | 0.000 | 0.41 | (0.27, 0.62) | | **0.000**^c^ | **0.41** | **(0.27, 0.62)** | | 195 | | 249 | |
| *BRIP1* | rs61169879 | 0.433 | 1.11 | (0.85, 1.45) | | 0.365 | 1.13 | (0.87, 1.48) | | 195 | | 249 | |
| *TRIM40* | rs9261484 | 0.095 | 1.31 | (0.96, 1.79) | | 0.118 | 1.28 | (0.94, 1.75) | | 195 | | 249 | |
| *MBNL2* | rs4771268 | 0.017 | 0.72 | (0.55,0.95) | | **0.015** | **0.72** | **(0.55, 0.94)** | | 195 | | 249 | |
| *FBRSL1* | rs11610045 | 0.675 | 1.09 | (0.74, 1.60) | | 0.699 | 1.08 | (0.73, 1.59) | | 195 | | 249 | |
| *DYRK1A* | rs2248244 | 0.519 | 1.10 | (0.83, 1.44) | | 0.463 | 1.11 | (0.84, 1.47) | | 195 | | 249 | |
| *RIMS1* | rs12528068 | 0.500 | 0.85 | (0.52, 1.38) | | 0.547 | 0.86 | (0.53, 1.41) | | 215 | | 236 | |
| *CD19* | rs2904880 | 0.541 | 1.14 | (0.75, 1.74) | | 0.443 | 1.18 | (0.77, 1.81) | | 195 | | 249 | |
| *SPTSSB* | rs1450522 | 0.596 | 0.93 | (0.70, 1.22) | | 0.607 | 0.93 | (0.71, 1.23) | | 195 | | 249 | |
| CI= confidence interval; HWE = Hardy-Weinberg Equilibrium; LOPD = late onset Parkinson’s Disease; MAF = minor allele frequency; OR = odds ratio; PD = Parkinson’s Disease; SNP = single nucleotide polymorphism.  ^a^*p* value, OR and 95% CI were obtained from risk analysis and refer to the risk allele.  ^b^ Adjusted for age and gender.  ^c^The statistical significances remained after using Bonferroni correction.  Bold character showed p<0.05 in the indicated model. | | | | | | | | | | | | | |

**Table S5**. Association of SNPs of candidate genes and odds ratio to EOPD risk

| Candidate Gene | SNP | HWE *p* value | MAF (case/control) | Allele | | | | | | | Dominant Model | | | | |
| --- | --- | --- | --- | --- | --- | --- | --- | --- | --- | --- | --- | --- | --- | --- | --- |
|  |  |  |  | Effect allele | *p^a^* | | | OR^a^ | 95%CI^a^ | | *p* | OR | | 95%CI | |
| *SNCA* | rs8180209 | 0.839 | 0.43/0.49 | A | 0.441 | | | 0.81 | (0.47, 1.39) | | 0.363 | 0.71 | | (0.33,1.49) | |
| *MCCC1* | rs2270968 | 0.973 | 0.38/0.29 | G | 0.147 | | | 1.46 | (0.87, 2.44) | | 0.145 | 1.72 | | (0.83, 3.57) | |
| *DLG2* | rs7479949 | 0.452 | 0.42/0.38 | C | 0.590 | | | 0.87 | (0.53, 1.44) | | 0.206 | 0.57 | | (0.24,1.36) | |
| *GBF1* | rs10748818 | 0.202 | 0.32/0.39 | G | 0.252 | | | 0.74 | (0.43,1.24 ) | | 0.291 | 0.68 | | (0.33, 1.39) | |
| *BRIP1* | rs61169879 | 0.627 | 0.44/0.39 | C | 0.391 | | | 1.24 | (0.76, 2.05) | | 0.609 | 1.21 | | (0.58, 2.54) | |
| *TRIM40* | rs9261484 | 0.948 | 0.22/0.25 | T | 0.648 | | | 0.87 | (0.48, 1.57) | | 0.612 | 0.83 | | (0.41, 1.70) | |
| *MBNL2* | rs4771268 | 0.200 | 0.38/0.45 | T | 0.244 | | | 0.74 | (0.44, 1.23) | | 0.705 | 0.87 | | (0.42, 1.80) | |
| *FBRSL1* | rs11610045 | 0.759 | 0.06/0.12 | A | 0.111 | | | 0.44 | (0.15, 1.24) | | 0.138 | 0.44 | | (0.15, 1.30) | |
| *DYRK1A* | rs2248244 | 0.390 | 0.38/0.35 | A | 0.721 | | | 1.10 | (0.66, 1.83) | | 0.848 | 1.07 | | (0.52, 2.20) | |
| *RIMS1* | rs12528068 | 0.313 | **0.01/0.08** | **T** | **0.034** | | | **0.15** | **(0.02, 0.53)** | | 0.069 | 0.15 | | (0.02, 1.16) | |
| *CD19* | rs2904880 | 0.338 | 0.13/0.10 | C | 0.559 | | | 1.25 | (0.59, 2.67) | | 0.389 | 1.43 | | (0.63, 3.25) | |
| *SPTSSB* | rs1450522 | 0.339 | 0.33/0.43 | A | 0.094 | | | 0.64 | **(**0.38-1.08**)** | | 0.088 | 0.53 | | (0.27, 1.10） | |
| Candidate Gene | SNP | Genetic Power | Dominant Model (adjusted)^b^ | | | | | Recessive Model | | | | Recessive Model (adjusted)^b^ | | | |
|  |  |  | *p* | OR | | 95%CI | | *p* | OR | 95%CI | | *p* | OR | | 95%CI |
| *SNCA* | rs8180209 | 0.091 | 0.383 | 0.63 | | (0.22, 1.78) | | 0.608 | 0.80 | (0.33,1.91) | | 0.633 | 0.94 | | (0.35, 2.52) |
| *MCCC1* | rs2270968 | 0.158 | 0.151 | 1.71 | | (0.82, 3.54) | | 0.506 | 1.47 | (0.47,4.59) | | 0.542 | 1.25 | | (0.61, 2.56） |
| *DLG2* | rs7479949 | 0.069 | 0.064 | 0.31 | | (0.09, 1.07) | | 0.402 | 0.42 | (0.05,3.24) | | 0.588 | 0.72 | | (0.07, 7.97) |
| *GBF1* | rs10748818 | 0.089 | 0.644 | 0.79 | | (0.28, 2.18) | | 0.411 | 0.60 | (0.17,2.05) | | 0.961 | 0.98 | | (0.37, 2.58) |
| *BRIP1* | rs61169879 | 0.095 | 0.844 | 1.11 | | (0.40,3.04) | | 0.359 | 1.49 | (0.64,3.51) | | 0.986 | 1.01 | | (0.32, 3.19) |
| *TRIM40* | rs9261484 | 0.070 | 0.414 | 0.66 | | (0.24, 1.80) | | 0.912 | 0.92 | (0.20,4.19) | | 0.322 | 0.30 | | (0.03,3.21) |
| *MBNL2* | rs4771268 | 0.088 | 0.828 | 0.89 | | (0.32, 2.47) | | 0.127 | 0.43 | (0.15,1.27) | | 0.178 | 0.39 | | (0.10,1.54) |
| *FBRSL1* | rs11610045 | 0.129 | 0.357 | 0.53 | | (0.14, 2.06) | | - | - | - | | - | - | | - |
| *DYRK1A* | rs2248244 | 0.053 | 0.461 | 0.68 | | (0.25, 1.89) | | 0.644 | 1.27 | (0.46,3.54) | | 0.598 | 1.46 | | (0.36, 3.92) |
| *RIMS1* | rs12528068 | 0.871 | **0.023** | **0.06** | | **(0.01, 0.68)** | | - | - | - | | - | - | | - |
| *CD19* | rs2904880 | 0.090 | 0.644 | 1.33 | | (0.40, 4.39) | | - | - | - | | - | - | | - |
| *SPTSSB* | rs1450522 | 0.063 | 0.385 | 0.64 | | (0.24,1.74) | | 0.331 | 0.58 | (0.20,1.73) | | 0.859 | 0.87 | | (0.19,4.04） |
| Candidate Gene | SNP | Additive model | | | | | Additive model (adjusted)^b^ | | | | | Number of sample tested | | | |
|  |  | *p* | OR | 95%CI | | | *p* | OR | 95%CI | | | Case | | Control | |
| *SNCA* | rs8180209 | 0.379 | 0.80 | (0.48, 1.32) | | | 0.411 | 0.76 | (0.39, 1.47) | | | 36 | | 249 | |
| *MCCC1* | rs2270968 | 0.153 | 1.47 | (0.87, 2.51) | | | 0.542 | 1.25 | (0.61, 2.56) | | | 36 | | 249 | |
| *DLG2* | rs7479949 | 0.493 | 1.26 | (0.66, 2.40) | | | 0.822 | 1.12 | (0.42, 3.03) | | | 36 | | 249 | |
| *GBF1* | rs10748818 | 0.057 | 0.57 | (0.32, 1.02) | | | **0.011** | **0.20** | **(0.06, 0.70)** | | | 36 | | 249 | |
| *BRIP1* | rs61169879 | 0.402 | 1.23 | (0.76, 2.01) | | | 0.893 | 1.05 | (0.55,1.98) | | | 36 | | 249 | |
| *TRIM40* | rs9261484 | 0.648 | 0.87 | (0.48, 1.57) | | | 0.293 | 0.63 | (0.27, 1.49) | | | 36 | | 249 | |
| *MBNL2* | rs4771268 | 0.263 | 0.76 | (0.46, 1.24) | | | 0.373 | 0.73 | (0.37, 1.45) | | | 36 | | 249 | |
| *FBRSL1* | rs11610045 | 0.124 | 0.44 | (0.16, 1.25) | | | 0.250 | 0.49 | (0.14, 1.66) | | | 36 | | 249 | |
| *DYRK1A* | rs2248244 | 0.714 | 1.10 | (0.65, 1.86) | | | 0.794 | 0.91 | (0.43, 1.90) | | | 36 | | 249 | |
| *RIMS1* | rs12528068 | 0.073 | 0.16 | (0.02, 1.19) | | | **0.024** | **0.06** | **(0.01, 0.70)** | | | 36 | | 249 | |
| *CD19* | rs2904880 | 0.566 | 1.24 | (0.59, 2.62) | | | 0.992 | 0.99 | (0.35, 2.81) | | | 36 | | 249 | |
| *SPTSSB* | rs1450522 | 0.088 | 0.63 | (0.36,1.07) | | | 0.462 | 0.75 | (0.35, 1.61) | | | 36 | | 249 | |
| CI= confidence interval; EOPD = Early Onset Parkinson’s Disease; HWE = Hardy-Weinberg Equilibrium; MAF = minor allele frequency; OR = odds ratio; PD = Parkinson’s Disease; SNP = single nucleotide polymorphism.  ^a^*p* value, OR and 95% CI were obtained from risk analysis and refer to the risk allele.  ^b^ Adjusted for age and gender.  Bold character showed p<0.05 in the indicated model. | | | | | | | | | | | | | | | |

# Table S6. Brain expression analysis stratified by SNP from Braineac database

| gene | rsID | exprID | aveALL | CRBL | FCTX | HIPP | MEDU | OCTX | PUTM | SNIG | TCTX | THAL | WHMT |
| --- | --- | --- | --- | --- | --- | --- | --- | --- | --- | --- | --- | --- | --- |
| SNCA | rs8180209 | **2777712** | 0.1400 | 0.2500 | 0.2500 | 0.9600 | 0.5700 | 0.6600 | 0.2100 | 0.3300 | 0.2200 | 0.2000 | **0.0170** |
|  |  | 2777756 | 0.2400 | 0.5500 | 0.8300 | 0.8600 | 0.5600 | **0.0230** | 0.2600 | 0.1200 | 0.5700 | 0.2900 | 0.9700 |
| MCCC1 | rs2270968 | **2707867** | **0.0004** | 0.5400 | **0.0380** | **0.0100** | 0.0990 | 0.6300 | **0.0066** | **0.0031** | 0.8400 | **0.0420** | 0.1200 |
|  |  | 2707825 | 0.0550 | 0.2700 | 0.2700 | **0.0014** | 0.3700 | 0.1600 | 0.7400 | 0.8600 | 0.3900 | 0.5000 | 0.8700 |
| DLG2 | rs7479949 | **3384715** | 0.1200 | 0.6300 | 0.5900 | 0.2600 | 0.0810 | **0.0380** | 0.2000 | 0.1700 | 0.0710 | 0.8200 | **0.0480** |
|  |  | 3384712 | 0.1600 | 0.1400 | 0.6300 | 0.3700 | 0.3800 | **0.0120** | 0.2100 | 0.7700 | 0.0770 | 0.6200 | **0.0430** |
|  |  | 3384707 | 0.1800 | 0.9200 | 0.8100 | 0.4800 | 0.5900 | 0.1500 | 0.2400 | 0.7100 | 0.3300 | 0.2800 | **0.0190** |
|  |  | 3384713 | 0.2300 | 0.3200 | 0.9000 | 0.2700 | 0.2900 | **0.0120** | 0.1600 | 0.8200 | 0.0930 | 0.7700 | 0.0520 |
| GBF1 | rs10748818 | **3261612** | 0.0600 | 0.6200 | **0.0260** | 0.7800 | 0.0560 | 0.2800 | 0.6000 | 0.4800 | 0.0610 | 0.8400 | 0.9600 |
|  |  | 3261582 | 0.1100 | 0.3800 | 0.0600 | 0.4000 | 0.4700 | 0.2400 | 0.1700 | 0.8400 | **0.0330** | 0.2600 | 0.3000 |
|  |  | 3261613 | 0.1300 | 0.3500 | 0.1500 | 0.4900 | 0.2900 | 0.7700 | 0.7900 | 0.8100 | **0.0150** | 0.9700 | 0.5200 |
|  |  | 3261610 | 0.1300 | 0.0990 | **0.0210** | 0.5800 | 0.2700 | 0.3000 | 0.5900 | 0.8200 | 0.7800 | 0.2500 | 0.7600 |
| MBNL2 | rs4771268 | **3497658** | 0.4300 | 0.5500 | 0.8400 | **0.0015** | 0.7100 | 0.9100 | 0.7400 | 0.5900 | 0.8200 | 0.7800 | 0.1800 |
|  |  | 3497641 | 0.7300 | 0.5300 | 0.4600 | 0.0680 | 0.5400 | 0.9800 | 0.7700 | 0.6000 | 0.1100 | 0.2700 | **0.0190** |
|  |  | 3497657 | 0.7800 | 0.9100 | 0.2200 | 0.1400 | 0.4600 | 0.2900 | 0.7800 | 0.5100 | 0.1600 | 0.7500 | **0.0087** |
| RIMS1 | rs12528068 | **2913161** | **0.0220** | 0.6500 | 0.6200 | **0.0440** | 0.3600 | 0.8400 | 0.5800 | 0.3400 | 0.8100 | 0.1400 | 0.3100 |
|  |  | 2913170 | 0.0520 | 0.4800 | 0.2200 | 0.6000 | 0.0770 | 0.6200 | 0.6600 | 0.5300 | 0.2700 | 0.5900 | **0.0130** |
|  | | | | | | | | | | | | | |

**Abbreviations:** CRBL:cerebellar cortex; FCTX: frontal cortex;HIPP: hippocampus;MEDU:medulla;OCTX: occipital cortex; PUTM : putamen;

SNIG:substantia nigra;TCTX:temporal cortex;THAL:thalamus;WHMT:white matter; Bold character showed p<0.05 in the indicated tissue.

**Table S7. GTEx analysis of single-tissue QTLs for rs8180209 and rs2270968**

|  | Gene Symbol | SNP ID | *p* Value | NES | Tissue |
| --- | --- | --- | --- | --- | --- |
| sQTLs | SNCA | rs8180209 | 4.40E-10 | 0.61 | Brain-Cortex |
|  |  |  | 3.00E-07 | 0.84 | Nerve - Tibial |
| eQTLs | MCCC1 | rs2270968 | 1.60E-12 | 0.16 | Nerve - Tibial |
|  |  |  | 5.60E-06 | -0.37 | Brain - Nucleus accumbens (basal ganglia) |
|  |  |  | 1.20E-05 | -0.29 | Brain - Cerebellum |
|  |  |  | 2.10E-05 | 0.33 | Brain - Putamen (basal ganglia) |
|  |  |  | 1.60E-12 | 0.16 | Nerve - Tibial |
|  |  |  | 5.60E-06 | -0.37 | Brain - Nucleus accumbens (basal ganglia) |
|  |  |  | 1.20E-05 | -0.29 | Brain - Cerebellum |

**Abbreviations:** sQTL:splicing quantitative trait loci; eQTL: expression quantitative trait loci
